# Sustained Attention in Intellectually Gifted Children Assessed Using a Continuous Performance Test

**DOI:** 10.1371/journal.pone.0057417

**Published:** 2013-02-25

**Authors:** Jiannong Shi, Ting Tao, Wei Chen, Li Cheng, Long Wang, Xingli Zhang

**Affiliations:** 1 Key Laboratory of Behavioral Science, Institute of Psychology, Chinese Academy of Science, Beijing, China; 2 Graduate University of Chinese Academy of Sciences, Beijing, China; 3 Faculty of Education, Beijing Normal University, Beijing, China; 4 Department of Learning and Philosophy, Aalborg University, Aalborg, Denmark; University of Melbourne, Australia

## Abstract

This study aimed to investigate two aspects of sustained attention in intellectually gifted children: first, the differences to intellectually average children; second, the differences between receiving standard education and enrichment education. Study 1 compared sustained attention between 24 intellectually gifted and 26 intellectually average children. The results showed that intellectually gifted children had better performance than their average peers, not only for the whole task but also on all indices. Study 2 compared sustained attention between 24 intellectually gifted children who received standard education and 24 intellectually gifted children who received enrichment education. The results showed that intellectually gifted children who received enrichment education performed better than those who received standard education. These findings are consistent with previous work and provide support for the implementation of enrichment education for intellectually gifted children.

## Introduction

Sustained attention is a form of attention that is responsible for the “continuous allocation of processing resources for the detection of rare events” [1] and refers to the capacity to maintain an engaging activity over a period of time. On the one hand, sustained attention requires an individual to focus his/her attention on ongoing events and to avoid missing anything. On the other hand, it requires an individual to be able to resist interference from the surrounding environment and to inhibit unnecessary impulses [2]. Therefore, sustained attention involves two different cognitive abilities: concentration and impulsivity controlling. It is the basis of high-level cognitive processing [3], [4].

Schweizer and Moosbrugger [5] investigated the relationship between sustained attention and intelligence. They found that sustained attention could significantly predict individuals’ performance on intelligence tests. Specifically, the control of attention provides unique contributions to both children’s and adults’ intelligence [6]. Liu and her colleagues’ event-related potential (ERP) research indicated that children with high IQs have better inhibition than do average children [7], [8], [9]. These findings suggest that the sustained attention of intellectually gifted children may be better than that of intellectually average children.

However, there are also conflicting findings. Many high ability students are referred due to problems with impulsivity, hyperactivity, and sustained attention [10], [11]. A study by Chae et al. [12] showed that 9.4% of the intellectually gifted children were diagnosed with ADHD, which is higher than the worldwide prevalence of ADHD from the general population or schools (5.29%) in Polanczyk et al.’s review [13]. Some studies have suggested that intellectually gifted children cannot focus attention on content in the classroom because the materials are too easy [14]. These findings suggest that the attention quality of intellectually gifted children may be worse than that of intellectually average children.

Summarize the opinions from the two perspectives, it can be found that the standpoint which support the intellectually gifted children have better sustained attention is more powerful than the opposite opinion. Combining the research in our lab [7], [8], [9], we insist that the standpoint intellectually gifted children have better sustained attention is more convincing. However, despite the different results of various studies, few studies have systematically investigated intellectually gifted children’s sustained attention. Which opinion is right? This is the first question we attempt to address in the present study by comparing the sustained attention of intellectually gifted children and intellectually average children. In this study, we hypothesize that the intellectually gifted children have better sustained attention than intellectually average children do.

The results from previous studies conflict with each other, some researchers insist that intellectually gifted children have better sustained attention [7], [8], [9], while others characterize the intellectually gifted children with attention problems [10], [11], [12]. Dose this controversy exist in all educational environments? This question is related to a controversial topic in gifted research: education of the gifted. Two education models have been developed for gifted education in China since 1978 [15]. The first model is accelerated education. In this model, students complete standard courses in a shorter time. The other model is enrichment education. In this model, schools provide various curricula for the children. As a new and developing education model, gifted education has received considerable attention from the public. One of the greatest concerns is its effect on intellectually gifted children. Previous research has investigated the effect of accelerated education on intellectually gifted children’s development and found that accelerated education had an accelerating effect on children’s information processing speed [16]. However, few studies have investigated the effect of the enrichment education model. The second question we want to investigate is the difference in sustained attention between intellectually gifted children who receive standard education and intellectually gifted children who receive enrichment education. There were few studies on the contrast of gifted children with other peers. This study could be a contribution to the literature and gifted and talented education.

As mentioned above, sustained attention is the ability to maintain an efficient level of response to a demanding task over a period of time [1], which is critical for daily activities and learning [17]. Performance on a task may vary over time as a function of arousal and the ability to sustain attention to the task [18], [19]. Parasuraman proposed that the deficit in performance over time is a result of the exhaustion of attention resources [20], and cognitive neuroscience studies have revealed that parallel reductions in cerebral blood flow are related to the decrement in vigilance [21], [22]. Some researchers have proposed that the decline in performance and vigilance over the duration of a task is the critical index of the impairment of sustained attention [23], [24]. As we all know, in the regular classroom, the gifted children often feel bored because of the unchallenging learning task, so they have no chance to practice the ability to sustain attention during a relative long time. As a consequence, they have much time to disrupt, make troubles, or self-amusing [11]. Therefore, in the present study, we divide the task into three stages to examine changes in sustained attention over time. The most frequently used method to measure sustained attention is the continuous performance task (CPT, [25], [26], [27], [28]) and all these studies showed good reliabilities and validity of the task. Continuous performance tasks require an individual to maintain vigilance while watching a long sequence of letters or numbers presented on a computer screen. Typically, the individual must press a button whenever one particular target letter or number appears. The 1–9 type CPT (number 1 followed by number 9) was used in this study.

The present study chose three groups of children: 1) intellectually average children who receive standard education, 2) intellectually gifted children who receive standard education, and 3) intellectually gifted children who receive enrichment education. The differences in sustained attention between intellectually gifted children and intellectually average children come from the comparison between groups 1 and 2. It was hypothesized that intellectually gifted children should outperform intellectually average children on the sustained attention task. The differences in sustained attention between intellectually gifted children who receive standard education and intellectually gifted children who receive enrichment education come from the comparison between groups 2 and 3. It was hypothesized that enrichment education would be more beneficial to develop sustained attention of intellectually gifted children than standard education.

## Study 1

### Materials and Methods

50 children participated in this study. 24 intellectually gifted children (mean age: 10.41±0.62 years old, 11 boys) and 26 intellectually average children (mean age: 10.62±0.53 years old, 14 boys) were assessed by the CPT. Intellectually gifted children were selected from a relative large sample (more than 1000 children). The age difference was not significant between the two groups, *t* = 1.29, *p*>0.05. There were 12 children in Grade 4 and 12 children in Grade 5 in the gifted group, and 8 children in Grade 4 and 18 children in Grade 5 in the average group. The Chi-Square test showed that the grade difference between the two groups was not significant, *χ^2^* = 1.92, *p*>0.05. We tested the intelligence of these two groups of children with the Raven Advanced Progressive Matrices (RAPM) Test before the formal experiment since the RAPM is a traditional instrument to test intelligence and it is relatively independent to verbal knowledge which is suitable to a large age span population. All of the intellectually gifted children’s intelligence scores were at Level 1, which means this group of children’s intelligence scores were in the top 5 percentile in their peers’ norm. All of the intellectually average children’s intelligence scores were at Level 2, which means this group of children’s intelligence scores were in the range of 25%–75% in their peers’ norm. All the children had normal or corrected normal vision and they were all right-handers.

The CPT task was completed on computers in classrooms. Each child completed a practice session until the examiner was confident that the child understood the task completely. The task consisted of 540 numbers (approximately 2 centimeters in size), which appeared on center of the computer screen, one at a time, for 200 ms. The ISI is 1000 ms. In order to analyze the sustained attention performance over time, we divided the task into three stages, each stage including 4 minutes. Participants were asked to press the space bar if the number 9 was preceded by the number 1. The event rate was 10% and this percentage was constant across the three stages. The total CPT task takes approximately 12 min for the participant to complete. The CPT generates several dependent measures including reaction time (RT) to correct responses, rate of omission error, rate of commission error, and the signal detection parameters, *d’* and β. Errors of omission occurred when participants failed to depress the spacebar on trials containing target numbers, which can reflect the degree of attention. Errors of commission occurred when participants depressed the spacebar on trials containing nontarget numbers, which can reflect inhibition ability. The signal detection measure *d’* is the distance between the signal distribution and noise distribution in standard score units, which reflects the participant’s perceptual sensitivity to targets. Higher *d’* values indicate higher amounts of signal detection relative to noise and suggest better discrimination between target and nontarget. β is a function of the ratio of target response to nontarget response, which represents an individual's response tendency. Some individuals are cautious and choose not to respond very often. Such individuals want to make sure they are correct when they give a response. Higher values of β reflect this response style. The emphasis is on avoiding commission errors. Other individuals respond more freely to make sure they respond to most or all targets, and they tend to be less concerned about mistakenly responding to a non-target. Lower values of β are produced by this response style.

Before participation parents and children signed informed consent forms. The study was approved by the Ethics Committee of the Institute of Psychology, Chinese Academy of Sciences.

### Results

#### Total comparison

Table 1 shows the descriptive data for the intellectually gifted and intellectually average children’s performance on the CPT task.

**Table 1:** Comparison of CPT performance between intellectually gifted and intellectually average children.

	RT	Rate of omission errors	Rate of commission errors	*d’*	β
Average(26)	388.91±75.49	0.28±0.20	0.0607±0.05	2.41±1.06	3.46±3.08
Gifted(24)	372.25±49.03	0.10±0.16	0.0319±0.05	3.91±1.44	7.98±13.44
T test	0.92	3.33**	2.17*	4.21***	1.61

* p<0.05,

** p<0.01,

*** p<0.001.

A t test was used to examine the differences between the intellectually gifted children and the intellectually average children on CPT task performance. The results showed that intellectually gifted children, compared with intellectually average children, had a significantly lower rate of omission errors and a lower rate of commission errors (see Table 1). Intellectually gifted children also had significantly higher sensitivity than intellectually average children did. The reaction time and judgment criterion between the two groups of children were not significant.

#### Stage comparisons

To analyze the changes in the process of the CPT task, we chose the rate of omission errors and the rate of commission errors as the indices in the stage comparisons.

**Figure 1:**
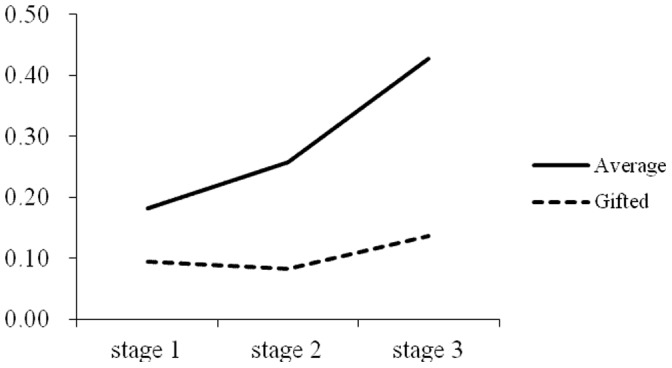
Trend of rate of omission error in the three stages of CPT between intellectually average and gifted children.

Repeated measures were used to test the differences among the three stages in the rate of omission errors for the two groups. For the intellectually average children, Greenhouse-Geisser analysis showed that the main effect of the stage on the rate of omission errors was significant, *F* (1.73, 43.28)  = 7.10, *p*<0.01, *η^2^* = 0.22. A paired comparison showed that the rate of omission errors in the 3^rd^ stage was significantly higher than the first two stages, *ps*<0.01. For the intellectually gifted children, the results showed that the main effect of the stage on the rate of omission errors was not significant, *F* (2, 22)  = 1.64, *p*>0.05, *η^2^* = 0.13. As shown in Figure 1.

**Figure 2:**
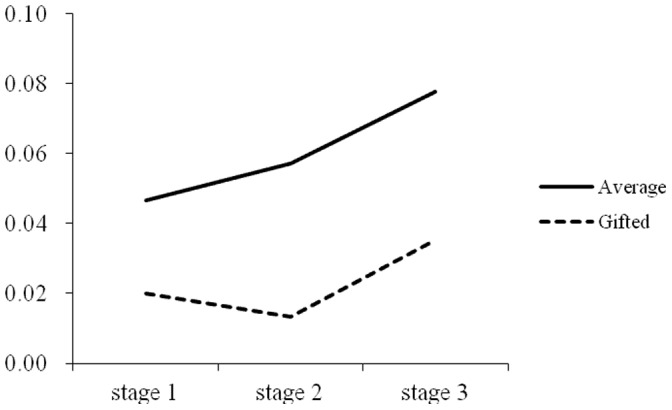
Trend of rate of commission error in the three stages of CPT between intellectually average and gifted children.

Repeated measures were used to test the differences among the three stages for the rate of commission errors for the two groups. For the intellectually average children, Greenhouse-Geisser analysis showed that the main effect of the stage on the rate of commission errors was marginally significant, *F*(1.86,46.42) = 2.69, *p* = 0.08, *η^2^* = 0.10. A paired comparison showed that the rate of commission errors in the third stage was significantly higher than in the first stage, *p*<0.05. For the intellectually gifted children, the results showed that the main effect of the stage on the rate of commission errors was not significant, *F*(2,22) = 1.97, *p*>0.05, *η^2^* = 0.15. As shown in Figure 2.

### Discussion

During the last few decades, researchers have focused on the cognitive abilities of intellectually gifted children. The results have shown that intellectually gifted children have obvious advantages in cognitive abilities over their intellectually average peers, such as faster information processing speed [29], better reasoning [30] and executive functions [31]. Given these superior cognitive skills, it makes sense that intellectually gifted children may also have a sustained attention advantage relative to their intellectually average peers. Findings from the current study supported this idea. As expected, intellectually gifted children had a lower rate of omission errors, suggesting that intellectually gifted children can concentrate their attention much better than intellectually average children [32], [33]. Intellectually gifted children also had a lower rate of commission errors, indicating that they have better inhibition ability and can control their impulses better than intellectually average children [32], [33]. Intellectually gifted children were also more capable of distinguishing targets from distracters than intellectually average children. However, there were no differences between intellectually gifted and average children in judgment criterion and reaction time.

According to Posner and Cohen’s classification of attention, goal-driven attention is referred to as endogenous attention, whereas stimulus-driven attention is referred to exogenous attention and is driven by external environmental events [34]. The sustained attention investigated in the current study was related to endogenous attention because the subjects followed particular instructions and the task followed general rules [35]. Compared to exogenous attention, endogenous attention depends more on the individual’s effort and his/her self-regulation ability. Prior work has shown that intellectually gifted children perform better on tasks that require cognitive control [7], [9], [36], and distraction inhibition abilities can predict individuals’ performance on cognitive tasks [37]. Furthermore, gifted students outperformed average students on metacognition [38]. These results refute the above-mentioned “inability to focus” phenomenon.

Intellectually average children’s performance, including rates of omission errors and rates of commission errors, showed an increasing tendency in the three stages, which was consistent with previous studies demonstrating that the participants’ performances declines over time when they are required to respond to rare stimulus events occurring in a sequence of standard stimulus events [39]. A convincing explanation is that a long period of vigilance requires considerable effort and places significant pressure on individuals [40], [41], [42]. However, for intellectually gifted children, the results were different: the rates of omission errors in the three stages were quite stable, and the rates of commission errors showed a declining trend in the second stage. These findings suggest that not only were intellectually gifted children able to maintain high concentration throughout the whole process, but they also became more careful in the process of the task and were able to inhibit their impulses better than average children.

From the perspective of sensitivity, which indicates the ability to distinguish targets from distracters [43], our results showed that intellectually gifted children had better sensitivity than intellectually average children did. This finding indicates that intellectually gifted children are more sensitive to targets and can distinguish targets from distracters better than intellectually average children. This finding confirms that intellectually gifted children have better sustained attention than their average peers.

Contrary to expectations, our results suggested that there was no difference between intellectually gifted and average children’s reaction times. One possible explanation for our failure to find differences between intellectually gifted and average children’s reaction times is that the task used in the current study was relatively easy. All of the children could provide timely responses, resulting in a ceiling effect of reaction time. Similarly, there was no difference between intellectually gifted and average children’s judgment criteria. Judgment criteria are influenced by individuals’ personality and reflect an ability that is relatively independent of intelligence. Conservative individuals tend to be strict on signal detection and usually have low judgment criterion values, whereas impetuous individuals are inclined to treat a stimulus as target and thus obtain high judgment criterion values [44]. The two groups of children did not differ in this characteristic.

In conclusion, intellectually gifted children have better attention quality than intellectually average children. Twin studies showed that general intelligence heritability is about 0.7 [45]. In view of the above, may be the most fundamental factor to explain the differences between intellectually gifted children and average children on their sustained attention performance is their biological differences on genes.

## Study 2

### Materials and Methods

48 children participated in this study. 24 intellectually gifted children (mean age: 10.67 years old, 11boys) were recruited from an experimental class that was specifically designed to offer enrichment education for gifted children nationwide. The gifted education system for these intellectually exceptional children is called the “Gifted Experiment Class”. It enrolls and educates 6-year-old children according to the normal course but provides much wider curricula and activities. Following this program, the children will have completed the entire primary school curriculum and are allowed to participate in the Middle School Entrance Examination.

Enrollment examinations of the gifted education system are held each year and recruit approximately 30 intellectually gifted children from more than 1000 candidates nationwide based on multiple criteria and methods. The main steps for recruitment and identification include an application, a primary screening test (focusing on general cognitive abilities, including reasoning, memory, and observation, etc.), a retest (focusing on five domains: cognitive abilities, creativity, learning ability, special talents, personality traits), further confirmation (more information about the children’s physical condition and their adaptation to new environments), behavioral observations in the actual educational environment and an investigation of the children’s actual and potential performance levels (children who had excellent performance on cognitive tests and academic achievement levels within the top 5 percentile passed this step) [46]–[47]. It should be noted that sustained attention is not included in the criteria used for the selection of intellectually gifted children who receive enrichment education in the present study.

The additional 24 intellectually gifted children who received standard education were the same as in Study 1. The two experiments share the same intellectually gifted children group because of the following two aspects: first, to reduce the differences between subjects to a minimum; second, there are really few intellectually gifted children according to the statistics, it is difficult to select many intellectually gifted children in different age groups.

We tested the intelligence of these two groups of children with the Raven Advanced Progressive Matrices Test before the formal experiment. All of the participants’ intelligence was at Level 1.

The task was the same as the task in Study 1. Before participation parents and children signed informed consent forms. The study was approved by the Ethics Committee of the Institute of Psychology, Chinese Academy of Sciences.

### Results

#### Total comparison

Table 2 shows the descriptive data for the two groups of intellectually gifted children’s performance on the CPT task.

**Table 2:** Comparison on CPT performance between standard education and enrichment education.

	RT	Rate of omission errors	Rate of commission errors	*d’*	β
Standard(24)	372.25±49.03	0.10±0.16	0.0319±0.05	3.91±1.44	7.98±13.44
Enrichment(24)	336.11±45.71	0.01±0.01	0.0050±0.01	5.32±0.70	4.29±6.85
T test	2.64*	2.91**	2.72*	4.33***	1.20

* p<0.05,

** p<0.01,

*** p<0.001.

A t test was used to examine the differences between the two groups of intellectually gifted children’s performance on the CPT task. The results showed that, compared to the intellectually gifted children who received standard education, intellectually gifted children who received enrichment education had significantly shorter reaction times (see Table 2). They also had lower rates of omission errors and lower rates of commission errors. Intellectually gifted children who received enrichment education had significantly higher sensitivity than intellectually gifted children who received standard education. The judgment criterion between the two groups of children was not significant.

Multivariate analysis of covariance analysis (MANCOVA) was performed to evaluate the contributions of the education to sustained attention index scores, age and Raven raw scores were entered as covariates. The results revealed significant main effects of education on rate of omission errors, *F*(1,47) = 9.553, *p* = 0.003, *η^2^* = 0.178; rate of commission errors, *F*(1,47) = 8.390, *p* = 0.006, *η^2^* = 0.160, and sensitivity, *F*(1,47) = 26.127, *p<*0.001, *η^2^* = 0.373, but no significant main effect on processing speed or beta scores. Children in enrichment special education condition performed better than children in regular education class on hit, inhibition and sensitivity. However, the results revealed no intelligence scores or age main effect on all the attention indices.

#### Stage comparisons

**Figure 3:**
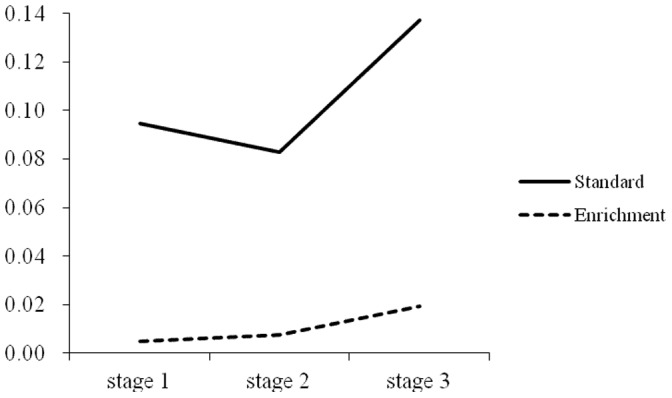
Trend of rate of omission error in the three stages of CPT between standard education and enrichment education.

Repeated measures were used to test the differences among the three stages on the rate of omission errors for the two groups. For the intellectually gifted children who received standard education, the results showed that the main effect of the stage on the rate of omission errors was not significant, *F*(2,22) = 1.64, *p*>0.05, *η^2^* = 0.13. For the intellectually gifted children who received enrichment education, the results showed that the main effect of the stage on the rate of omission errors was not significant, *F*(2,22) = 1.31, *p*>0.05, *η^2^* = 0.11. As shown in Figure 3.

**Figure 4:**
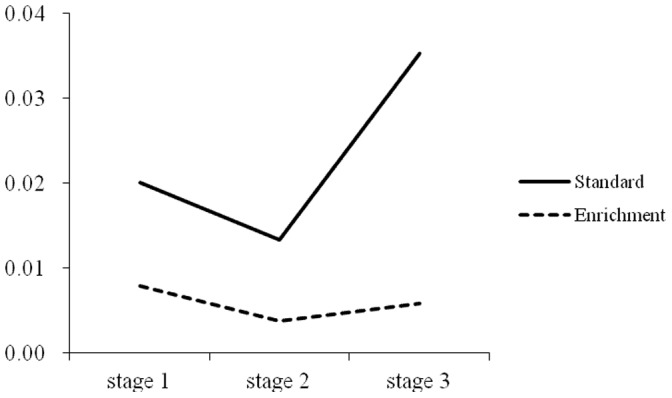
Trend of rate of commission error in the three stages of CPT between standard education and enrichment education.

Repeated measures were used to test the differences among the three stages in the rate of commission errors for the two groups. For the intellectually gifted children who received standard education, the results showed that the main effect of the stage on the rate of commission errors was not significant, *F*(2,22) = 1.97, *p*>0.05, *η^2^* = 0.15. For the intellectually gifted children who received enrichment education, Greenhouse-Geisser analysis showed that the main effect of the stage on the rate of commission errors is not significant, *F*(1.83,42.08) = 2.41, *p* = 0.10, *η^2^* = 0.10. However, paired comparison showed that the rate of commission errors in the second stage was significantly lower than in the first stage, *p*<0.05. As shown in Figure 4.

## Discussion

As mentioned above, gifted education is a new and developing education model. One of the greatest, public concerns about gifted education is its effect. Researchers in gifted education have proposed that a student’s educational program should be determined by his/her needs and interests [48], which is the goal of gifted education. Thus, it is logical to infer that intellectually gifted children who receive enrichment education have better sustained attention quality than intellectually gifted children who receive standard education. The results of study 2 confirmed this hypothesis.

Compared to intellectually gifted children who received standard education, intellectually gifted children who received enrichment education also had a significantly lower rate of omission errors and commission errors, suggesting that intellectually gifted children who receive enrichment education can concentrate their attention better and longer, and they also can control their impulses better than intellectually gifted children who receive standard education [32], [33]. Furthermore, intellectually gifted children who receive enrichment education can distinguish targets from distracters more accurately than intellectually gifted children who receive standard education.

However, after controlling the IQ score,the result of study 2 revealed that there were no significant main effect of education on response time or beta scores. Response time was highly related to the innate ability [49], the result suggested that the two groups have comparable processing speed. Some twin study findings suggest the strong relationship between the speed of processing and IQ (phenotypic correlations:−.31 to −.56 in males; genotypic correlations: −.45 to −.70). Beta score reflected the individual response tendency, the results suggested that the two groups had no difference in judgment criteria, which influenced by individuals’ personality and reflect an ability that is relatively independent of intelligence [44]. Overall, our findings suggest that intellectually gifted children who receive enrichment education have superior sustained attention. It is important to note that in the three stages of the CPT task, both groups of children maintained a stable rate of omission errors. Although intellectually gifted children who received standard education had lower rates of omission than intellectually gifted children who received enrichment education during the entire process of the task. Like intellectually gifted children who received enrichment education, they could maintain stable vigilance in the three stages of the task. This result indicates the high concentration quality of intellectually gifted children, which was consistent with the results of study 1. It is also notable that intellectually gifted children who received standard education only displayed a non-significant decreasing tendency in the rate of commission errors in the second stage. However, for intellectually gifted children who received enrichment education, the rate of commission errors in the second stage was significantly lower than in the first stage. This finding suggests that intellectually gifted children who received enrichment education became more careful in the process of the task and could inhibit their impulses better than intellectually gifted children who received standard education. It was reasonable to propose that enrichment education provides a more suitable environment for intellectually gifted children to develop better sustained attention, because the challenging curricular program and the contents of the learning satisfy the need of these children’s information processing; on the contrary, because of the unchallenging curricular, intellectually gifted children in normal education just need less time to learn the materials which normal children need much more time to learn. In this case, the intellectually gifted have nothing to do in most of the class time, which causes their ADHD-like behaviors among the normal peers [11], [50]. In summary, we can infer that intellectually gifted children who receive enrichment education have better attention quality than intellectually gifted children who receive standard education do. It is logical to infer that enrichment education can improve the quality of intellectually gifted children’s sustained attention. How can enrichment education do this? The educational environment around intellectually gifted children is often quite basic and does not meet their needs. In a regular classroom, the teaching content is basic and unchallenging. Therefore, it is not surprising that these children feel bored. Enrichment education can provide a variety of courses that are more interesting and more challenging for intellectually gifted children. In this environment, intellectually gifted children can focus their attention naturally. Furthermore, teachers have fewer complaints about intellectually gifted children’s attention in this educational environment. Previous studies have compared the speed of information processing between children who received standard education and gifted education, and these results showed that the reaction time of intellectually gifted children who received gifted education was significantly faster than that of the children who received standard education at every age [16], [51], [52]. Combined with the results of the current study, it is logical to infer that the educational environment of an intellectually gifted child plays a significant role in the development of his/her cognitive ability.

The long-term effects of being identified and/or educated as a gifted student have been confirmed by the Study of Mathematically Precocious Youth. This study investigated various outcomes, including attaining a PhD, obtaining a tenure track position at a top university, the number of patents secured, income and life quality [52], [53], [54], [55], [56]. Lubinski and colleagues [55] found that students who were identified in talent searches earned PhDs at 50 times more than the rate of those in the general population. Similarly, Lubinski and colleagues [54] investigated academic and other accomplishments and found that individuals identified as gifted at age 13 using the SAT were comparable to those in the top graduate programs of their respective fields. It is obvious that it is vital to identify and accommodate the gifted. However, there may be some question of whether the quality of gifted education is much better than standard education. It is not surprising that children who receive gifted education develop better than children who receive standard education. If intellectually average children can receive gifted education, they may develop as well as intellectually gifted children. Undoubtedly, this suggestion is reasonable. However, according to the work of Cheng and colleagues, intellectually gifted children can benefit more from practice [50]. Therefore, it is reasonable to expect that intellectually gifted children can benefit more from gifted education. Furthermore, standard education cannot meet intellectually gifted children’s needs, but it can satisfy intellectually average children very well.

Given these findings, the implementation of enrichment education for intellectually gifted children is important and necessary.

## Conclusions

Synthesizing the two studies, we can clearly see that, compared with intellectually average children, intellectually gifted children have three notable features: first, intellectually gifted children can concentrate their attention better than their average peers; second, with a familiar task, intellectually gifted children can restrain their impulses better than intellectually average children, they become more cautious in the process and they perform better; and third, intellectually gifted children have better sensitivity than intellectually average children do. They have better ability to distinguish a target from distracters, which is an index of high-quality attention. These three features may reflect the essential differences in sustained attention between intellectually gifted children and intellectually average children.

There are also some deficiencies in the present study. First, gender difference is a controversial issue in the field of research on gifted children. Extensive discussions have examined gender differences in gifted children. In this study, we did not address this issue because of the limited subject quantity. Second, the present study only selected children aged 10 to compare in response to some reasons: first, intellectually gifted children under enrichment education aged 10 have received more than three years enrichment education, we can investigate the effect of this kind of gifted education program; second, we mentioned that there are two kinds of gifted education in China, enrichment education and accelerated education. Some of the gifted children who received enrichment education may choose to go to other schools to receive accelerated education to save time. In order to guarantee the number of the participants, we only chose children aged 10. Third, motivation in the process of completing the task was not considered. Intellectually gifted children may keener in accomplishing the task than intellectually average children due to the high parents’ expectation and acted as they were in a competition. However, in the process of explaining how to do the task, the experimenters tried to encourage the children do their best to complete the task. In future studies, we will follow up with the subjects in the present study and choose additional subjects to explore gender differences and the trajectory of sustained attention of intellectually gifted children. Furthermore, we will attempt to use the ERP technology to determine the brain mechanisms involved in intellectually gifted children’s sustained attention.
